# A ‘Life-Style Choice’ or a Philosophical Belief?: The Argument for Veganism and Vegetarianism to be a Protected Philosophical Belief and the Position in England and Wales

**DOI:** 10.1007/s10991-020-09273-w

**Published:** 2021-01-16

**Authors:** Paul McKeown, Rachel Ann Dunn

**Affiliations:** Northumbria School of Law, Northumbria University, City Campus East 1, Newcastle upon Tyne, NE1 8ST UK

**Keywords:** Veganism and vegetarianism, Protected philosophical beliefs, Employment, Animal welfare

## Abstract

The recent judgment in *Casamitjana Costa v The League Against Cruel Sports* in England and Wales held that ethical veganism was a protected philosophical belief under employment law. In contrast, vegetarianism was found not to be a protected philosophical belief in *Conisbee v Crossley Farms Limited and others*. The authors argue that the Employment Tribunal misunderstood the notion of vegetarianism when deciding that it was a ‘life-style choice’. There are different kinds of vegans and vegetarians, each with their own way of practising the philosophy which influences how they live their life. Not all people who follow a meat-free diet should be afforded this protection, and it depends on whether their belief is one which is determined by certain factors, such as animal welfare and environmentalism, rather than for health purposes. The authors explore the arguments and analysis in the above employment cases, coming to the conclusion that the tribunals oversimplified what it means to hold values such as veganism and vegetarianism, failing to understand the differences between different classifications and sub-groups when coming to a decision. The different kinds of vegans and vegetarians and their characteristics are outlined, before determining whether this should constitute protection under employment law, protecting individuals from discrimination. The situation in the USA and Canada regarding this issue is very different, and there are parallels drawn with attempting to establish veganism or vegetarianism as a religion, and where they could benefit from the recent decision in England and Wales. Finally, this paper concludes that ethical and environmental veganism and vegetarianism should both qualify as protected philosophical beliefs, but other kinds may fall short of what is required to satisfy the requirements under law.

## Introduction

Veganism is by no means a new concept, but has become more prominent in recent years, particularly in the UK. Statistics show that in 2018 the UK had the highest number of new vegan products launched,^1^ which is understandable when the number of vegans has risen to 600,000 in 2019, from 150,000 in 2014, with about half of UK vegans converting in 2018.^2^ Other research found that vegetarianism is the most popular non-meat diet, followed by pescatarian and then vegan, with an estimation that 6.7 million British adults currently follow a meat-free diet, but with an indication that this will rise to 12 million by the end of 2020.^3^ The reasons for becoming vegetarian or vegan^4^ vary, including to improve health, the environmental impacts and, the main motivation, animal welfare concerns.

Despite the rising popularity of veganism and vegetarianism, those who practice as vegans or vegetarians can be subjected to discrimination. There are reasons as to why individuals are opposed to the vegan diet, and actively argue against it, with views that ‘predation is natural and that animals often kill and eat each other.’^5^ Others see it as an attack on their autonomy of choice or a conflict with the omnivore’s majority beliefs.^6^ Some of the difficulties vegans report in their life is eating out, finding vegan substitutes for clothing and household products, and feeling isolated from omnivore family and friends.^7^ Further, vegans and vegetarians face stereotypes and prejudices on a regular basis, such as the belief they want to convert omnivores to veganism and are judgmental toward them, that they are hippies and all are animal activists.^8^ Some even hide their beliefs in the workplace, for the fear of prejudice, or avoid conversations about their beliefs at mealtimes, wanting to avoid confrontation.^9^

Though vegetarians and vegans may have such a strong belief, they are not more biased than meat eaters, but as Adams argues, they ‘do not benefit as meat eaters from having their biases actually approved of by the dominant culture’.^10^ Further, it has been trivialised, or seen as a ‘distraction’ from other important aspects of history, and judged as irrelevant to other topics such as sexism and racism.^11^ This discrimination, or bias, does happen though, and studies have found that vegans and vegetarians are subjected to attitudes which are equivalent or more negative than common prejudice target groups, including significantly more negatively than black people, and overall vegans were viewed more negatively than vegetarians.^12^ This was not, however, in all aspects of life, and they were not subject to less willingness to be hired for a job or rented accommodation compared to other target groups, including immigrants and atheists.^13^ This kind of bias even has a name: veganphobia. This bias and discrimination can affect individuals in the work place, for example being forced to use animal products in the workplace,^14^ or their taxes going toward systems which abuse animals, ‘forcing vegans to contribute to something they strongly disagree with.’^15^

In January 2020, an Employment Tribunal in England held ethical veganism to be a philosophical belief and therefore a protected characteristic pursuant to the Equality Act 2010.^16^ As such, ethical vegans may gain protection from discriminatory treatment. In contrast however, an Employment Tribunal previously held that vegetarianism was not capable of satisfying the requirements of being a philosophical belief.^17^ The authors will argue that these cases oversimplified concepts of veganism and vegetarianism. In particular, the authors will analyse what it means to hold beliefs in veganism and vegetarianism, and how the tribunal failed to understand the differences between different classifications and sub-groups when coming to a decision.

This article begins by defining religion, philosophical beliefs and creeds, looking at the legal position in the England and Wales, US and Canada. A discussion of the philosophy of veganism and vegetarianism follows, looking at the work of Francione specifically, before exploring the influencing factors of becoming, and kinds of, a vegan or vegetarian. It is analysed whether veganism and vegetarianism are protected philosophical beliefs, exploring recent case law in the England and Wales, and whether they should be protected. Finally, this article concludes which classifications of veganism and vegetarianism should both qualify as protected philosophical beliefs, though, some classifications may fall short of what is required to satisfy the requirements under law.

## Defining Religion, Philosophical Beliefs and Creeds

*Everyone has the right to freedom of thought, conscience* and religion; *this right includes freedom to change his* religion or *belief*, and freedom, either alone or in community with others and in public or private, *to manifest his* religion or *belief in teaching, practice*, worship and *observance*.^18^{emphasis added}

The text above acknowledges the universal human right to thought, conscience and beliefs. This right is further enshrined into international law through Article 18 of the International Covenant on Civil and Political Rights 1966. The movement to eradicate discrimination on the grounds of religion and belief is further re-enforced through the UN Declaration on the Elimination of All Forms of Intolerance and of Discrimination Based on Religion or Belief. Whilst Article 1(1) of the aforementioned Declaration re-iterates the language of ‘thought, conscience and religion’, Article 1(2) states, ‘No one shall be subject to coercion which would impair his freedom to have a religion or *belief of his choice*.’{emphasis added}

Article 9 of the European Convention of Human Rights (ECHR) also enshrines the right to freedom of thought, conscience and religion, whilst within the European Union, Article 10 the Charter of Fundamental Rights of the European Union embeds freedom of ‘thought, conscience and religion’ within member states.^19^ Whilst there have been no cases determining the meaning of thought, conscience and religion within the context of the Charter of Fundamental Rights of the European Union, the matter has been considered in relation to the ECHR.

Religion is not defined within the text of Article 9 nor in the European Court of Human Rights case law.^20^ This is deliberate, as any definition would need to be ‘both flexible enough to embrace the whole range of religions worldwide…and specific enough to be applicable to individual cases—an extremely difficult, indeed impossible undertaking.’^21^ However, Article 9 ECHR has been held to apply to ‘the “major” or “ancient” world religions’, ‘new or relatively new religions’ and ‘various coherent and sincerely-held philosophical convictions’.^22^

Freedom of belief, including secular beliefs, is well established in international law, as summarised above. However, whilst international law recognises the freedom of religion and belief, the protection of these freedoms needs to be afforded at domestic level for individuals to meaningfully avail themselves to protection of these freedoms as the discrimination will often emanate from individuals or private companies rather than the State. Further, where freedom of religion and belief are protected at domestic level, it is for national authorities to interpret the extent of the protection afforded based upon their jurisdictional definition of religion and belief, or whatever wording each jurisdiction may utilise. The difficulties on establishing an international consensus on the definition of religion and belief can be seen with certain ‘religions’ such as the Church of Scientology.^23^

Within England and Wales, religion and belief are a protected characteristic pursuant to the Equality Act 2010.^24^ Whilst claims for contravention of the ECHR can only be brought against public authorities,^25^ courts and tribunals must interpret legislation, so far as is possible, in a way which is compatible with the ECHR.^26^ As such, the definition of religion and belief is a ‘broad definition in line with the freedom of thought, conscience and religion guaranteed by Article 9’.^27^

Religion is defined under the Equality Act 2010 as ‘any religion and a reference to religion includes a reference to a lack of religion.’^28^ Thus, religion is defined as itself. The definition encompasses traditional religions such as Buddhism, Christianity, Hinduism, Islam and Judaism but also smaller religions such as Rastafarianism or Paganism, as long as it has a clear structure and belief system.^29^ The approach appears to be that whilst religion is not defined in England and Wales, the courts and tribunals will know it when they see it.

Section 10(2) of the Equality Act 2010 defines ‘belief’ as ‘any religious or philosophical belief and a reference to belief includes a reference to a lack of belief.’^30^ In determining what constitutes a ‘philosophical belief’, the Explanatory Notes to the Equality Act 2010 provide some guidance stating the belief ‘must be genuinely held; be a belief and not an opinion or viewpoint based on the present state of information available; be a belief as to a weighty and substantial aspect of human life and behaviour; attain a certain level of cogency, seriousness, cohesion and importance; and be worthy of respect in a democratic society, compatible with human dignity and not conflict with the fundamental rights of others.’^31^ Further, the belief must have a similar status or cogency to a religious belief.^32^

There is an inherent problem in providing a statutory definition for philosophical beliefs due to the widely varying beliefs that individuals may hold and which of these beliefs should be protected. As with religion, it has therefore been left to the courts to define whether a particular belief should be protected and indeed the guidance set out above was implied or introduced by reference to the jurisprudence.^33^

In the United States, freedom of religion is established by the First Amendment of the Constitution stating that ‘Congress shall make no law respecting an establishment of religion, or prohibiting the free exercise thereof…’^34^ Whilst the First Amendment regulates the role of the Government in legislating on religion, it does little to protect individuals from being discriminated against by other individuals or private organisations. At Federal level, Title VII of the Civil Rights Act of 1964 (Title VII) prohibits employment discrimination on the grounds of, inter alia, religion.^35^ In contrast to the Equality Act 2010, Title VII only refers to ‘religion’, not ‘religion and belief’. It is therefore necessary to consider the definition of religion for the purposes of Title VII. Religion is defined broadly to include ‘all aspects of religious observance and practice, as well as belief…’^36^ Whilst it includes all traditional religions, it also encompasses unorganised and less common systems of belief.^37^ The US Equal Employment Opportunity Commission (EEOC) have adopted the definition of religion given by the US Supreme Court in *United States v Seeger* and therefore, it is religious if it is “a sincere and meaningful belief that occupies in the life of its possessor a place parallel to that filled by…God.”^38^ As such, religious beliefs include theistic and non-theistic beliefs, although beliefs are not protected merely because they are strongly held.^39^ In *United States v Meyers*,^40^ the court set out the following factors to assist in determining whether a belief is religious: Ultimate Ideas; Metaphysical Beliefs; Moral or Ethical System; Comprehensiveness of Beliefs; and Accoutrements of Religion.^41^ The latter factor considers the following: Founder, Prophet, or Teacher; Important Writings; Gathering Places; Keepers of Knowledge; Ceremonies and Rituals; Structure or Organization; Holidays; Diet or Fasting; Appearance and Clothing; and Propagation.^42^

The court recognised that no one factor is dispositive, and instead the factors should be seen as ‘criteria’ that if ‘minimally satisfied’ should include the beliefs with the term religion.^43^ However, ‘[p]urely personal, political, ideological, or secular beliefs probably would not satisfy enough criteria for inclusion.’^44^ Examples of beliefs falling outside the definition of religion include: nihilism; anarchism; pacifism; utopianism; socialism; libertarianism; Marxism; ‘vegetism’; and humanism.^45^ Such an effect this has had on gaining protection for veganism and vegetarianism in the US, some have argued for the establishment of a Church of Animal Liberation, to provide a religious organization for those who wish to seek protection and accommodation for their beliefs in animal rights.^46^

In contrast to the United States Constitution, the Canadian Charter of Rights and Freedoms (the Canadian Charter) enshrines ‘conscience and religion’ as well as ‘thought, belief, opinion and expression’ as fundamental freedoms.^47^ Moon posits that ‘distinguishing religious beliefs/practices from secular beliefs/practices’ in Canada resolves the problem ‘which has bedevilled the American courts’.^48^ However, the Canadian Charter only applies to Governments, not disputes between individuals, businesses and other organisations.

The Canadian Human Rights Act does prohibit discrimination on the grounds of religion.^49^ Whilst the Act only applies to federally regulated activities, each province and territory has its own anti-discrimination laws, adopting differing approaches to religion and creed.^50^ The Supreme Court of Canada has defined religion broadly ‘as a particular and comprehensive system of faith and worship’ which ‘tends to involve the belief in a divine, superhuman or controlling power.’^51^ However, this definition did not include ‘secular, socially based or conscientiously held’ beliefs.^52^ The Supreme Court of Canada summarised the definition stating:

…religion is about freely and deeply held personal convictions or beliefs connected to an individual’s spiritual faith and integrally linked to one’s self-definition and spiritual fulfilment, the practices of which allow individuals to foster a connection with the divine or with the subject or object of that spiritual faith.^53^

In 2015, the Ontario Human Rights Commission updated their ‘Policy on preventing discrimination based on creed’. Whilst creed was previously interpreted to mean religion,^54^ in the updated policy, creed was defined to ‘also include non-religious belief systems that, like religion, substantially influence a person’s identity, worldview and way of life.’^55^ Whilst there is no single definition, the factors identifying a creed within this policy are; it is sincerely, freely and deeply held; it is integrally linked to a person’s identity, self-definition and fulfilment; it is a particular and comprehensive, overarching system of belief that governs one’s conduct and practices; it addresses ultimate questions of human existence, including ideas about life, purpose, death, and the existence or non-existence of a Creator and/or a higher or different order of existence; and it has some “nexus” or connection to an organization or community that professes a shared system of belief.^56^

In considering the various definitions used, it is clear that religion, philosophical beliefs and creed have been interpreted broadly. However, the various jurisdictions have been reluctant to include secular beliefs, which would include veganism and vegetarianism, within the definition of religion. Whilst the position is still unclear with regards to the United States, which will be considered below, a secular belief system is more likely to be protected when legislation expressly differentiates between religion and other beliefs. This can be seen in international law, specifically within the context of the ECHR, within England and Wales protecting philosophical beliefs, and in Ontario, Canada developing the definition of creed as distinct from religion.

There is also a distinct similarity between the factors determining whether a belief constitutes a philosophical belief in England and Wales, or a creed in Ontario, Canada as shown in Table 1 below.^57^ This suggests that philosophical belief and creed are synonymous with one another.

**Table 1:** Factors determining a philosophical belief in England and Wales or a creed in Ontario, Canada

‘Philosophical belief’ under the Equality Act 2010	‘Creed’ pursuant to Ontario Human Rights Commission Policy on preventing discrimination based on creed
Must be genuinely held	Is sincerely, freely and deeply held
Be a belief and not an opinion or viewpoint based on the present state of information available	Is integrally linked to a person’s identity, self-definition and fulfilment
Be a belief as to a weighty and substantial aspect of human life and behaviour	Is a particular and comprehensive, overarching system of belief that governs one’s conduct and practices
Attain a certain level of cogency, seriousness, cohesion and importance	Addresses ultimate questions of human existence, including ideas about life, purpose, death, and the existence or non-existence of a Creator and/or a higher or different order of existence

## Protection Afforded to Philosophical Beliefs and Creeds

The extent of protection afforded by anti-discrimination legislation varies between jurisdictions. In England and Wales, the Equality Act 2010 makes it unlawful to discriminate against an individual on the grounds of a protected characteristic. The Act applies to numerous areas such as the provision of services,^58^ housing,^59^ employment,^60^ education,^61^ and associations.^62^ As such, individuals are protected from discrimination in many aspects of their lives. There is a wide breadth of this protection in comparison to anti-discrimination legislation in other jurisdictions. For example, Title VII only relates to the employment field, and therefore does not offer protection from discrimination in other areas such as the provision of services. Further, it only applies where an employer employs fifteen or more employees.^63^ As such, there are significant limitations in the protection afforded under Title VII. However, there are various statutes in each State protecting freedom of religion. The Canadian Human Rights Act applies in the areas of the provision of goods and services,^64^ commercial and residential premises^65^ and employment.^66^ The Canadian Human Right Act is limited to Federally regulated activities although each Province has their own anti-discrimination legislation.

The Equality Act 2010 protects individuals from different types of discrimination, namely: direct; indirect; harassment; and victimisation. Whilst there will be instances of individuals experiencing direct discrimination^67^ i.e. not being employed because of their vegan beliefs; in many instances, it is likely that an employer or service provider simply does not cater for their requirements. For example, a failure to provide a vegan meal option or the requirement to wear a uniform manufactured with non-vegan products. The issue is not that the individual is vegan per se, it is the manifestation of those vegan beliefs, i.e. their dietary requirements or refusal to wear clothes manufactured from animal products, which places the individual at a disadvantage in comparison to non-vegans.

Under Article 9 ECHR,^68^ the freedom of religion and belief is an absolute right. However, the right to manifest those beliefs may be limited where it is ‘prescribed by law and…necessary in a democratic society in the interests of public safety, for the protection of public order, health or morals, or for the protection of the rights and freedoms of others.’^69^

The Equality Act 2010 protects individuals from discrimination because of a manifestation of their religion or belief through indirect discrimination where a ‘provision, criterion or practice’ is applied equally, whether or not individuals share the protected characteristic but places individuals who share the protected characteristic at a particular disadvantage in comparison to those who do not share the characteristic, placing the individual at a disadvantage.^70^ However, unlike direct discrimination, indirect discrimination can be justified if it can be shown to be a ‘proportionate means of achieving a legitimate aim.’^71^

With reference to the above examples, a failure to provide a vegan food option or the imposition of a uniform policy is a ‘provision, criterion or practice’ which would clearly put an individual who is vegan at a disadvantage compared with a non-vegan. The question therefore is whether an employer or service provider can establish that the ‘provision, criterion or practice’ was a proportionate means of achieving a legitimate aim. Section 19 of the Equality Act 2010 should be read compatibly with Article 9(2) ECHR. What is proportionate will differ in every case, so the more serious the impact of the policy, criterion or practice, the greater the justification will need to be for implementing the policy.

Courts and tribunals will balance business needs against the impact on the group disadvantaged. In applying this to the above scenarios, it is likely that the provision of a vegan meal option or allowance of a suitable alternative uniform will suffice to negate any claims for indirect discrimination. However, an example where the provision, criterion or practice may be proportionate is a requirement for a vegan shop worker to handle notes, which are made with animal products, as the business requires the handling of cash. Further, it may be appropriate to discipline an employee for proselytising about their vegan convictions if it violates the dignity of other workers.^72^

Individuals will also be protected from harassment which is defined as unwanted conduct related to a relevant protected characteristic which has the purpose or effect of violating their dignity, or creating an intimidating, hostile, degrading, humiliating or offensive environment.^73^ As such, individuals will be protected from bullying or mockery due to their philosophical beliefs and/or the manifestation of those beliefs.

Similar types of discrimination are included within Title VII prohibiting disparate treatment,^74^ disparate impact,^75^ a failure to accommodate religion,^76^ and harassment.^77^ The right to reasonable accommodation for religion protects those manifesting their belief, and offers a greater degree of protection than indirect discrimination within England and Wales as there is no requirement to establish a disproportionate impact on a group, merely the individual.

Under the Canadian Human Rights Act, direct discrimination (or disparate treatment) is unlawful within the prescribed areas,^78^ as is harassment^79^ and retaliation^80^ (similar to victimisation). However, a discriminatory policy or practice is only applicable to employment matters albeit there is no justification defence.^81^ Once again, different Provinces have their own anti-discrimination legislation.

## Philosophy of Vegetarianism and Veganism

The concept of vegetarianism and veganism has been explored in the literature around animal ethics, with philosophers such as Peter Singer^82^ and Tom Regan^83^ arguing for the abstaining of eating meat and potentially other animal derived products, albeit with differing philosophical underpinnings to come to this conclusion. None are more aligned to the principles of ethical veganism, or abolitionist veganism, than Gary Francione, who argues that this diet rejects any use of animal products, and is ‘the moral baseline for the animal rights movement.’^84^ He rejects any other reasons, or approaches, for animal rights, stating that environmental vegans, or philosophers such as Singer who may allow for the use of animals in certain circumstances, are not really vegans, and do not see veganism as a “philosophy of living” but merely a lifestyle.^85^ Thus, those who are serious about the animal rights movement are those who adopt this philosophy in all aspects of their life, and accept that animal lives have moral significance, rejecting their current commodity status.^86^ Francione takes his abolitionist view further than food, and argues that we should not have companion animals (though he himself has dogs he sees as ‘refugees’) and should cease to bring domesticated non-human animals into existence, purely for our benefit.^87^ This is the same as in *Casamitjana Costa,* where the Claimant did not live with a companion animal.

Ethical and environmental vegans ‘make their choices in line with their core values. They want to live in alignment with their beliefs.’^88^ Thus, in order to do so, they change the way in which they live their life, adhering to a set of rules which looks to minimise the detrimental impacts a non-vegan life can have on sentient beings and the environment. This can be seen as the practice Ahimsa, the vow of non-injury, the prime practice in Jainism. Apparently, all ‘Jainas are strict vegetarians, living solely on one-sense beings (vegetables) and milk products. Alcohol, honey, and certain kinds of fig are also prohibited, because they are said to harbour many forms of life.’^89^ Thus, it can be seen how veganism has developed from the practice of Ahimsa, taking the vow of non-injury further and modernised it, to incorporate other forms of nonviolence, such as cosmetic and medical products and clothing, which has come at the expense of violence to animals. It is important to note that the philosophy of Ahimsa also extends to non-injury to the environment, and has been argued to be capable of addressing current bio-diversity issues the planet is facing.^90^ Thus, though animal rights advocates, such as Francione, argue that ethical veganism is the only way to project animals to a higher moral and legal standing and produce consistent behaviour, it seems that environmental vegans and their beliefs are also encapsulated by the teachings of Ahimsa and the philosophy on non-injury. The teachings of Ahimsa were discussed in *Casamitjana Costa,* and it was highlighted that the Claimant lived his life in line with these teachings and beliefs, which will be discussed in more detail below.

There are arguments which seek to undermine the philosophy and practice of ethical veganism, however, and they should be addressed. Some people argue that ethical veganism does not, for example, minimise the suffering of animals, due to how many animals are killed in fields from the ploughing of fields and harvesting of vegetables and grains.^91^ Therefore, ‘a vegan diet doesn’t necessarily mean a diet that doesn’t interfere with the lives of animals.’^92^ This is true, and in the process of gathering food, whether on an industrial or home-grown organic scale, there will be other beings harmed in the process. Francione argues that this is unavoidable, and all human actions have consequences, some of them adverse. This is no reason, though, to argue against the use of animals and the intentional harm humans cause to them.^93^ This is the thinking that helped the Claimant in *Casamitjana Costa,* and the fact that he would only use animal by-products where there was no other alternative, and only after exhausting all reasonable steps ‘to ensure that his consumption contributes as little as possible to the suffering and/or exploitation of sentient beings no matter how remote that is.’^94^ This was to the extent of food products, such as figs, which he believed would cause harm, and is consistent with the practices of Ahimsa. Whilst the argument of unintentional harm is valid, it does not affect the beliefs held by ethical vegans.

## Veganism and Vegetarianism Dissected

There is a clear distinction between veganism and other kinds of non-meat diets, and there is a large variety of diets available. For example, pescatarians do not eat meat, but eat fish and other seafood, as well as other animal derived products, like milk, eggs and cheese. Vegetarians do not eat meat, including ingredients such as gelatine, but eat other animal derived products. Vegans, on the other hand, do not eat any animal derived products, including honey. It is defined by the Vegan Society as ‘a way of living which seeks to exclude, as far as is possible and practicable, all forms of exploitations of, and cruelty to, animals for food, clothing or any other purpose.’^95^ This has been a gradual shift in movement from a promotion of a vegetarian diet to a vegan diet, starting in the early 1900’s in the UK, where the Vegetarian Society broke off into two strands with some asking for vegetarians to refrain from eating eggs, milk and products made with them.^96^ Leneman states that the main argument for a vegan diet was ‘always the cruelty, inseparable from the acquisition of dairy products, and the linkage of the meat and dairy industries.’^97^ There were, however, other arguments including health and consistency with the philosophy.^98^ Thus, it is necessary to explore why individuals choose to go vegetarian or vegan, before going on to discuss in depth the different kinds of diets. There are other influencing factors for going vegetarian or vegan, including religion and as part of the feminist movement, but for the purposes of this article and focusing on protected philosophical beliefs, only factory farming, animal welfare, the environment, health, and working conditions in slaughter houses will be discussed.

### The Farming Industry

It is no longer a secret that the farming industry has industrialised and, as a result, intensive farming practices have ensued.^99^ Harrison acknowledges that as ‘people become richer, they tend to want more meat, with the result that more and more animals are being farmed for food.’^100^ Since the publication of *Animal Machines* in 1964,^101^ which first exposed the suffering inflicted on animals in factory farms, there have been some significant changes, particularly in the EU and the UK. For example, veal crates, which restrict the movement of calves who are usually tied by their necks, have been banned in the UK since 1990,^102^ and the EU brought in a ban in 2006.^103^ These crates, however, are still legal in countries such as the US. Whilst there have been some significant improvements, there are certain pressures contributing to intensive farming, such as population growth, urbanization, economic growth and nutrition transition.^104^ As a result of this demand, the farming industry has developed to incorporate increasing use of technology, (such as artificial insemination, and advanced feeding systems), suffered structural changes (influenced by factors such as cost reduction), and lessened the restraints of local resources, (such as the rise of supermarkets).^105^

These intensive farming practices are having a negative impact on animal welfare, the environment, public health, and the welfare of slaughter house workers.

### Animal Welfare

Compassion in World Farming state that 74 billion animals are reared for food each year, with 50 billion reared for food through intensive farming on factory farms.^106^ There are many animals which are reared for food, including, but not limited to, cattle, pigs, sheep and chickens, and those who are reared on factory farms face dismal living conditions before slaughter. As stated above, this was brought to light in the UK through the book *Animal Machines*,^107^ but there are others who have shed light on the conditions such as Peter Singer in *Animal Liberation*,^108^ PETA and Compassion in World Farming. The conditions in which animals live on these farms are too extensive to fully outline in this article, but a small discussion will illustrate how they contribute to the factors of becoming a vegan or vegetarian and the philosophy of such practices.

Compassion in World Farming state that there are more than 50 billion chickens reared annually for food, either broiler chickens or egg-laying hens,^109^ making them the most farmed animal in the world. For example,^110^ in the UK there were 157,000 cattle slaughtered in April 2020,^111^ compared to 104 million broiler chickens in the same month.^112^ On these farms, chickens are often kept in battery cages,^113^ no larger than a single sheet of A4 paper, which restricts them from exhibiting normal behaviour patterns, such as nesting or foraging for food.^114^ Further, due to crowding, egg-laying hens feather-peck each other, leading to injuries. As a result, it is practice to de-beak the bird, removing a part of the beak with a hot blade and no aesthetic.^115^ Broiler chickens are kept in barns with no natural lighting, and are barren apart from food and water. They are bred to grow intensively, resulting in their legs not being able to carry their weight and suffer from leg disorders. Many die in these sheds from excessive heat, heart attacks, and ammonia pollution produced by their droppings.^116^ This is one example of the animal welfare issues on factory farms, but there are many conditions deemed to be ‘common practice’ which result in immense suffering for various animals, such as cows, pigs and sheep.

Vegans, particularly ethical vegans, take the point of animal welfare further, and their philosophy is influenced by other animal welfare issues outside of factory farms. For example, strict ethical vegans do not eat honey, as they believe the harvesting of honey does not abide by their definition of veganism, and it exploits the bees involved in the process.^117^ The honey industry can involve specific breeding of honey bees to increase productivity and clipping the wings of queen bees to prevent them setting up a colony elsewhere. Further, there are many animal welfare issues associated with industries such as fur,^118^ cosmetic and medical testing,^119^ and animals used in entertainment.^120^ Clearly our use of animals raises ethical and moral questions, those which vegans, and perhaps to a lesser extent vegetarians, have answered with abolishing any involvement with such industries and choosing to live a life which does not contribute to such welfare violations. After all, the consumer is somewhat responsible for the continuation of such suffering of animals, and to reduce this responsibility DeGrazia argues we should live by the following moral rule: ‘make every reasonable effort not to provide financial support to institutions that cause extensive unnecessary harm.’^121^

### Environmental Factors

It is becoming more prominent that current farming practices, whether that be for food or other industries such as factory and fur farming, are causing an impact on our environment. The Pew Commission reported that there are three main causes of environmental degradation resulting from intensive farming: large volumes of animal waste, the disposal of these materials and unsustainable water usage and soil degradation associated with feed.^122^ There are other issues, including factory farming releasing large amounts of toxic air emission, such as ammonia, causing a risk to public health.^123^ Waste can also disturb the environment, and the enjoyment of it, in other ways, such as the odours from poultry facilities attracting flies and rodents, which can carry disease.^124^

Land degradation is a common result of unsustainable agricultural practices, and ‘refers to a temporary or permanent decline in the productive capacity of the land of its potential for environmental management.’^125^ This not only damages the environment, but can also affect national food supply, trade and malnutrition.^126^ The decrease in the usage of this land due to degradation reduces productivity, and therefore has an economic consequence for farmers, pushing farmland into natural habitats, causing land destruction. This impacts on climate change, loss of biodiversity and depletion of water resources.^127^ With regard to climate change, it is becoming increasingly clear that greenhouse gas emissions are severely contributed to by animal agriculture, and agriculture is ‘directly responsible’ for approximately 20% of greenhouse gasses produced by human-generated emissions.^128^ By lowering the number of intensively farmed animals, we can lower the impact on climate change. Further, it is predicted that climate change will make it harder to produce enough food needed to meet growing population demands.^129^ It is important to recognise that some non-meat diets can also negatively contribute to climate change, and there needs to be consideration still as to which products are consumed in order for the positive impacts to be fully realised.^130^

Lastly, there is ‘easily enough grain protein, if used sensibly, to feed every human on earth.’^131^ Not only could this grain protein be used to feed humans rather than animals, but the amount of energy (calories) livestock feed consumes is almost 43%, and animal products return 29%, making it an inefficient system.^132^ The amount calories used to feed animals could, therefore, be used directly as human food, creating an annual calorie need for over 3.5 million people.^133^

### Health

The impact of intensive farming on rural communities has been researched, noting detrimental effects on physical health, mental health, and social health. For example, Horrigan et al. note how pollution from factory farms harms the health of workers and residents, causing respiratory diseases such as asthma, bronchitis and organic dust toxic syndrome.^134^ This is caused by the environmental impacts, discussed above.

Factory farms also use antibiotics and hormones, as a way to limit diseases in livestock and promote growth and weight gain.^135^ However, this has caused antimicrobial resistance in the environment, and more drug resistant infections have increased in humans. In fact, it is becoming such an issue that the World Health Organisation has asked that farmers and the food industry refrain from routinely using antibiotics.^136^ Some countries have, and the EU banned the use of antibiotics for growth production in 2006.^137^ The reasons for this are the serious health implications this resistance can have for human health. For example, LA-MRSA identified in pig production is a health risk for the farmers and veterinarians who come into contact with the animals and, though it is unclear what the public health relevance is of consuming contaminated meat, it has been found in pork and meat products.^138^

As well as antimicrobial resistance, other concerns with factory farming include the transmission of zoonotic diseases from animals to humans, becoming increasingly more common and prevalent. Morse et al. outline how no pandemic pathogens have been predicted before appearing in humans,^139^ though over 70% of emerging infection diseases in humans are zoonotic.^140^ For example, the current Covid-19 pandemic is thought to have started in a wet-market in Wuhan, China, which sold wildlife as meat.^141^ These issues aren’t isolated to wet-markets, however, and there are many opportunities for animal diseases to jump to humans. Morse et al. identify three stages to assess pandemic potential, the first being no human infection, but factors can contribute to transmission between hosts, expanding within its population, and be transmitted to other non-human populations, increasing likeliness of transmission to humans. Moving of livestock and transportation of wildlife for food can all contribute to a stage 1 emergence.^142^ The Food and Agriculture Organisation have stressed that transport systems are ‘ideally suited for spreading disease, as the animals commonly originate from different herds or flocks and are confined together for long periods in poorly ventilated, stressful environments.’^143^ However, there are also cases of animal–animal and animal-human disease being spread from animals transported for use in research, horses moved for equestrian competitions, and in the exotic pet trade.^144^

Another health aspect to take into consideration is the health benefits which come with a low meat, or non-meat, diet. It has been shown that high meat consumption, particularly of red meat, can cause cancers, heart disease, type 2 diabetes, and abstinence from meat products, even a vegetarian diet, can significantly lower mortality rates from these illnesses.^145^ It is important to highlight, however, that there are some health negatives from following these diets, however, particularly with veganism which can cause some deficiencies if not substituted adequately.^146^

### Workers

There is less focus on the working conditions in factory farms and slaughterhouses, but it is becoming more discussed. In the Global South, such as Kenya, very few slaughterhouses provide their workers with protective equipment and hand washing facilities, and a high level of illnesses is reported.^147^ In western societies, there are still dangers associated with the job, mainly due to speed at which slaughterers have to work in order to meet targets. Injuries include musculoskeletal injuries, such as carpal tunnel syndrome, and more life-threatening injuries, often with the knives used to cut through bone.^148^ Workers have reported being crushed by animals falling out of apparatus or kicked by them as they struggle.^149^ There are even deaths caused by poisonous fumes being inhaled while cleaning a blood-collection tank.^150^

Accounts of emotional and mental health of slaughterers being affected have emerged, leading to alcohol related problems and even suicide.^151^ This has been confirmed by research, which found that slaughterers during the initial stages of their employment had frequent vivid dreams about their work, feeling guilt and shame. In order to deal with these emotions, workers adopt psychological defences and become emotionally detached from their work. This can lead to expressions of anger which spill over into the home as abuse and violence.^152^ Though there is no evidence of a vegan of vegetarian choosing to adopt the belief because of this issue, with emerging research and information it may become an influencing factor.

There is also an issue with the wages of slaughterers which have fallen below that of an average manufacturing wage,^153^ to maintain production speed despite more meat being produced, and workers not being allowed to go to the toilet and wearing adult diapers or refraining from urinating and causing health issues.^154^ The conditions for workers is clearly a very important issue, and one that Muller says to ignore ‘is to ignore a key corner of the intersectional labyrinth that is the pursuit of social justice.’^155^

### Different Branches of Veganism and Vegetarianism

How people practice veganism does differ, and a study found that those who ‘created and abided by personal, idiosyncratic definitions of veganism, which were considerably less strict and often included dairy products and honey’, compared to those who followed the rules set by the Vegan Society.^156^ As stated above, there are various reasons why one becomes a vegan, and Greenebaum separates vegans into three district categories: health, environmental and ethical vegans.^157^ The authors would actually add another kind of vegan which, whilst has links to other groups of vegans, has its own distinct set of characteristics: the humanitarian vegan. There was not any previous research found, which explored this kind of vegan, though some may mention it as an influencing factor and link it to environmental factors, and it may be because there is not enough awareness of these issues. Nonetheless, the authors felt it important to include. Not all of the issues discussed in the previous sections will influence an individual to become vegan, but can be factors in the decision to follow the diet and lifestyle.

What becomes more complicated is where vegetarianism fits into the argument, with some people putting ethical vegetarianism and veganism in the same category.^158^ Further, there was less literature which focused solely on ethical vegetarianism, and some which referred to ethical veganism as ‘strict vegetarianism’.^159^ The main difference is that, whilst both abstain from eating meat, not all vegetarians will refrain from eating other animal derived products (e.g. eggs) or using animal derived products (e.g. leather), but some will. The differences and similarities between the groups can be different for each individual, and it is not simple to state that vegans have a stronger ethical belief than vegetarians do. Veganism is a relatively newer concept, when one considers the history and development of vegetarianism, and veganism, outside of religion, began as a concern for animal welfare.^160^ There are different strands of vegetarianism, such as lacto-ovo vegetarians, who do not eat meat, but eat diary and eggs, or ovo vegetarians, who include eggs in their diet, but not meat or milk/milk products.^161^ The underpinning beliefs of animal welfare and environmental practices may be the same, but the practice of those beliefs may vary,^162^ and it is not only veganism, but also vegetarianism, which can be seen as ‘being about defining the self, defining who one is, what sort of being one is, what it is to be human and the relationship one has with the non-human…’^163^ Thus, due to the consistent grouping of these terms, they are discussed together, but some of the differences are highlighted throughout the discussions

**Fig. 1:**
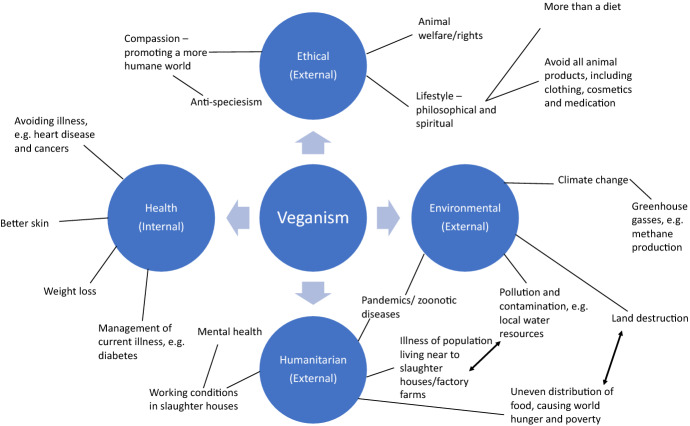
Different kinds of vegans and influencing factors

Figure 1 displays the different kinds of vegans, and the general characteristics which contribute to their belief and identity based on previous research which has explored these influencing factors:

Munroe’s research argues that ethical vegetarianism is part of the animal welfare movement, a kind of DIY activism, ‘used by activists to publicize an issue as well as to disrupt life in its immediate vicinity.’^164^ To disrupt this life even further, ethical vegans do not use any animal derived products at all, including those in cosmetics or clothing. Greenebaum states an ethical vegan is someone who ‘adopts a vegan diet for moral, ethical and political reasons. The diet forms only part of a lifestyle that is structured around philosophy of animal rights.’^165^ Further, her research found that ethical vegan participants saw veganism as a belief and ‘“liv[ing] in a connected way to the world around you.”’^166^ Their philosophy was so strong that the ethical vegan participants did not like the approach of the health vegans, stating that the purpose of giving up animal products is to no longer harm animals, not to lose weight. Maxim groups the health and environmental rationales together, stating they are merely dietary preferences.^167^ The authors would not necessarily agree with this, as ethical, environmental and humanitarian vegans all have an external factor which drives their belief in veganism and dictates how they live, whether this be the treatment of animals or the influence on climate change. A health vegan is the only kind which has an internal driving force, usually the need to control, or prevent, an illness and for the health and body image benefits.

These findings are consistent with other studies, where the driving factor for becoming a vegan was animal welfare issues, followed by health considerations.^168^ Mann’s study also found that an influencing factor was that a vegan diet can feed more people, linking into arguments of humanitarian veganism outlined above, as well as the impacts on the environment.^169^ Further, a quarter of participants reported being vegetarian, before making the change to veganism, some as a purposeful transition into veganism, and some ultimately moving that way to further eliminate animal cruelty.^170^ Interestingly, two vegans in this study who stated they did not follow a vegan lifestyle solely became vegans for health purposes.^171^ There are some studies which show, however, that the driving factor for becoming a vegan was for health reasons.^172^ Thus, the reasons for becoming a vegetarian or vegan are complex, multi-faceted, and can evolve and change over time.

## Is Veganism and Vegetarianism a Protected Philosophical Belief?

Vegan convictions have long been held to fall within the scope of Article 9 ECHR following the 1993 decision in *W v the United Kingdom*.^173^ However, whether veganism and vegetarianism are protected philosophical beliefs has only recently been tested in the domestic courts and tribunals of England and Wales.^174^

In 2011, an Employment Tribunal in *Hashman v Milton Park (Dorset) Limited t/a Orchard Park* found belief in the sanctity of life to fall within the definition of philosophical belief.^175^ Beliefs in the sanctity of life incorporated ‘beliefs in the value of life or veganism, environmentalism and animal rights activism.’^176^ As such, it was clearly arguable that veganism and vegetarianism could be regarded as philosophical beliefs.

There was discussion during the passage of the Equality Bill through Parliament as to whether veganism and vegetarianism were included within the definition of philosophical belief, with Baroness Warsi stating:

…the weight of case law meant that only serious and important beliefs would be included as a religious or philosophical belief for the purposes of the law…to include cults and other lifestyle choices such as veganism and vegetarianism is to make something of a farce of the debates that we had.^177^

This statement highlights that the inclusion of veganism and vegetarianism within the definition of philosophical belief was controversial, equating veganism and vegetarianism to a ‘cult’. The Equality and Human Rights Commission initially included veganism as an example of a belief attaining protection under the Equality Act stating in draft guidance:

A person who is a vegan chooses not to use or consume animal products of any kind. That person eschews the exploitation of animals for food, clothing, accessories or any other purpose and does so out of an ethical commitment to animal welfare. This person is likely to hold a belief which is covered by the Act.^178^

The Government did not share the view that veganism was covered although accepted the decision was ultimately for the courts to determine.^179^ The above cited example of veganism as a protected belief did not appear in the final guidance.

In 2019, in *Conisbee v Crossley Farms Limited and others*,^180^ an Employment Tribunal rejected that vegetarianism was capable of being a philosophical belief within the meaning of the Equality Act 2010. The Claimant, a barman/waiter, alleged that he had been bullied during the course of his employment because he was a vegetarian.^181^ The Claimant’s vegetarianism stemmed from his belief ‘that the world would be a better place if animals were not killed for food.’^182^ It was accepted that the Claimant was a vegetarian, that he had a genuine belief in his vegetarianism,^183^ and that the practice of vegetarianism was worthy of respect in a democratic society and not incompatible with human dignity.^184^ However, the tribunal concluded that the belief in vegetarianism was an opinion and view point,^185^ which could not be described as a weighty and substantial aspect of human life and behaviour, merely a life style choice.^186^ Finally, the tribunal concluded that the Claimant’s belief did not attain the required level of cogency, seriousness, cohesion and importance as vegetarians could adopt the practice for many different reasons such as lifestyle, health, diet, concern about the way animals are raised or indeed, personal taste.^187^ Further, the tribunal stated that the belief did not have a similar status or cogency to that of religion.^188^

In contrast to *Conisbee*, ethical veganism was held to be a philosophical belief in the subsequent Employment Tribunal case, *Casamitjana Costa v The League Against Cruel Sports*,^189^ decided in January 2020. The Claimant alleged that he had been dismissed after he wrote to colleagues advising them that their pensions were being invested in non-ethical funds, specifically pharmaceutical and tobacco companies known to engage in animal testing, and the only ethical fund offered by their employer offered worse rates of return than other ethical funds on the market.^190^ The Claimant alleged that the investments were ‘directly contradictory to the reason for the existence and values of the organisation.’^191^ Further, the investment also contravened the Claimant’s beliefs as set out below. It is important to highlight that the judgments in both *Casamitjana Costa* and *Conisbee* are First Instance decisions, and therefore not binding. The decisions in these judgments may not be followed in other Employment Tribunals and may be reversed if appealed.^192^

The Claimant in *Casamitjana Costa* did not eat any animal products nor did he purchase any animal products, including products tested on animals; his beliefs had other consequences for his life.^193^ Examples include:

- He would not allow non-vegan food to be brought into his home by any other person;
- He would not consume food where he believed its production harmed animals in any way;
- He would not attend any spectacle involving live animals, including zoos, circuses and animal races;
- He would not live with an animal companion;
- He would avoid social gatherings where non-vegan food was to be served;
- He would not date a non-vegan nor share his property with anyone who was not vegan;
- If his destination was within an hour’s walking distance, the Claimant would normally walk rather than use public transport to avoid accidental crashes with insects or birds; and
- He would usually pay for purchases using with a credit card or coins, avoiding as far as possible notes which have been manufactured using animal products.^194^

It is interesting to contrast the rationale for the difference in finding vegetarianism not to be a philosophical belief whilst ethical veganism is regarded a philosophical belief.^195^

### Opinion and Viewpoint

In *Conisbee*, the tribunal held that the ‘Claimant’s belief in vegetarianism was his opinion and viewpoint *in that the world would be a better place if animals were not killed for food*.{emphasis added}’.^196^ In *Casamitjana Costa*, the tribunal found that ethical veganism was more than an opinion and viewpoint stating:

…ethical veganism carries with it an important moral essential. That is so even if the Claimant may transgress on occasions. It is clear it is founded upon a longstanding tradition recognising *the moral consequences of non*-*human animal sentience* which has been upheld by both religious and atheists alike. Furthermore, there is no doubt that the Claimant personally holds ethical veganism as a belief. He has clearly dedicated himself to that belief throughout what he eats, where he works, what he wears, the products he uses, where he shops and with whom he associates. It clearly is not simply a viewpoint, but a real and genuine belief and not just some irrational opinion.^197^{emphasis added}

It is difficult to reconcile the reasoning given in these two cases. In both cases, the Claimants established a belief in how humans used animals; the difference seemingly the extent of that belief and its manifestation. In *McClintock v Department of Constitutional Affairs,*^198^ the Employment Appeal Tribunal differentiated a ‘belief’ from an ‘opinion or viewpoint’ stating:

…to constitute a belief there must be a religious or philosophical viewpoint in which one actually believes; it is not enough “to have an opinion based on some real or perceived logic or based on information or lack of information available.”^199^

In *Conisbee*, the Claimant’s belief that the world would be a better place if animals were not killed for food appears to be just that: a belief. The judgment does not make any reference to any logic or information which would support the Claimant merely having an opinion or viewpoint. The Claimant in *Conisbee* asserted that it was ‘wrong and immoral to eat animals’.^200^ As such, there is an analogy with the reasoning in both *Hashman v Milton Park (Dorset) Limited t/a Orchard Park*^201^ and *Casamitjana Costa*. The common feature of all these cases is the belief in the relationship between humans and animals. However, it is perhaps the case that sanctity of life encompasses beliefs in the value of life or veganism, environmentalism and animal rights activism, thus impacting upon the individual’s life to a greater extent than merely adopting a vegetarian diet. Similarly, veganism, particularly in the case of *Casamitjana Costa*, also has a significant impact upon the individual’s daily life. This, however, suggests that the test in some way relies upon the extent and manifestation of the belief and therefore the test has been wrongly applied, as the law does not provide that the manifestation belief has to be extreme or applicable to every aspect on the individual’s life.

### Lifestyle Choice

In *Conisbee*, whilst acknowledging the Claimant’s belief in vegetarianism was an ‘admirable sentiment’, the tribunal determined that it was a lifestyle choice that could not ‘altogether be described as relating to weight and substantial aspect of human life and behaviour.’^202^ It is clear that the Claimant’s vegetarianism in *Conisbee* did not impact upon his daily life as much as the that of the Claimant’s veganism in *Casamitjana Costa*, therefore it is necessary to consider what differentiates a belief from a mere lifestyle choice.

It is not necessary for a philosophical belief to ‘govern the entirety of a person’s life’.^203^ Indeed, vegetarianism was suggested as an example of such a belief.^204^ Again, it is difficult to reconcile the reasoning provided in each of the cases. The tribunal accepted in *Casamitjana Costa* the ‘relationship between humans and other fellow creatures is plainly a substantial aspect of human life’^205^ and therefore it should follow that someone who does not eat animal flesh due to their belief that it is ‘wrong and immoral’ should also satisfy this aspect of the test as it is more than a mere lifestyle choice.

### Cogency, Seriousness, Cohesion and Importance

In *Conisbee*, the tribunal rejected the notion that vegetarianism attained a certain level of cogency, seriousness, cohesion and importance.^206^ The tribunal compared vegetarianism with veganism stating ‘the reason for being a vegetarian differs greatly…unlike veganism where the reasons for being a vegan appear to be largely the same.’^207^ The tribunal assert that there are many reasons to become vegetarian; lifestyle, health, diet, animal welfare and personal taste.^208^ However, the tribunal state that vegans do not accept the practice of eating animal flesh or products under any circumstances, due to a distinct concern about the way animals are reared and a ‘clear belief that killing and eating animals is contrary to a civilised society and also against climate control.’^209^ A similar statement was made in *Casamitjana Costa* acknowledging ethical veganism as attaining cogency, cohesion and importance describing ethical veganism as:

a way of life which seeks to exclude as far as possible and practical all forms of exploitation and cruelty to animals for food, clothing or any other purpose and by extension promotes the development and use of animal free alternatives for the benefit of humans, animals and the environment.^210^

In *Conisbee*, the tribunal were dismissive of the notion that vegetarianism achieved a similar status or cogency to that of religion. The judgment provides little in the way of reasoning, merely stating it was not enough to have a belief relating to an important aspect of human life or behaviour.^211^ Interestingly, the tribunal in *Casamitjana Costa* did not expressly consider whether veganism attained a similar status or cogency to that of religion, albeit this finding could be implied from the discussion of veganism’s root in Ahimsa.^212^ Ahimsa was not discussed greatly in the judgment, but was highlighted that the concept of veganism is rooted in it, that it is the belief of causing no harm or injury, and that the Claimant followed these principles as a firm believer.

The tribunal has misunderstood the concepts of vegetarianism and veganism, using the terminology generally where the concepts are, in reality, more nuanced. As discussed above, there are different classifications and subsets of vegetarians and vegans. Each classification or subset of veganism or vegetarianism should be considered to determine whether they satisfy the criteria for protection as a philosophical belief.

Whether veganism or vegetarianism are protected in the United States remains debateable. Schwartz outlines how ethical veganism as religious discrimination is not a new idea in the United States, but has ‘never fully made its way to the courts on its merits.’^213^ To the authors’ knowledge, the only decided cases on the matter have found against veganism being recognised as a religion. In 2002, *Friedman v Southern California Permanente Medical Group*,^214^ determined that veganism was not a ‘religious creed’ within the meaning of meaning of the California Fair Employment and Housing Act. In 2006, a Californian Federal Court found that veganism was not a religion for the purpose of the First Amendment in relation to a prisoners request for a vegan diet.^215^ However, the EEOC has indicated that that they consider that a ‘strict vegetarian’ does fall within the definition of religion for the purposes of Title VII.^216^ Further in 2012, the United States District Court Southern District Of Ohio Western Division found ‘it plausible that [the Plaintiff] could subscribe to veganism with a sincerity equating that of traditional religious views.’^217^ In both cases, the matter settled before a court determination and therefore, whilst these cases have not established that ethical veganism is a religion, they have also not finished the conversation of it in the courts.

The question of veganism and vegetarianism have not been tested before any Canadian court within the authors’ knowledge. In the 2012 case, *Ketenci v Ryerson University*,^218^ the Human Rights Tribunal of Ontario did not find it necessary to consider whether ethical veganism fell within the definition of creed pursuant to the Ontario Human Rights Code.^219^ The tribunal determined that the applicant had no reasonable prospect of establishing that she was discriminated against because of her beliefs in ethical veganism.^220^

The Ontario Human Rights Commission did consider veganism within their consultation on the definition of creed. Drawing upon the work of Labchuck and Szytbel, it was suggested that confining the definition of creed to religion could result in the absurdity of differently sourced beliefs in ethical veganism being protected differently.^221^ Labchuck provided the example of: (1) a Jain follower, who is vegan for religious reasons; (2) a practising Christian who sees veganism as a religious duty: (3) A Christian who is vegan, but is a vegan for secular moral relating to animal welfare; and (4) An atheist who is an ethical vegan for strictly secular moral reasons.^222^ Labchuck argued that excluding secular beliefs from the definition of creed would result in the ‘apparent logical absurdity’ that protection would only apply to the first two examples despite all being equally committed to the same ethical vegan beliefs.^223^

As a consequence of the above consultation, the ‘Policy on preventing discrimination based on creed’^224^ was updated to explicitly state creed included non-religious beliefs. Based upon the consultation, there is clearly an argument that creed includes ethical veganism.

It appears the definition of creed will be tested before the Human Rights Tribunal of Ontario to determine whether it includes ‘ethical veganism’ in the case of *Knauff v Ministry of Natural Resources*. This case concerns a firefighter, with a belief in ethical veganism, alleging a chronic lack of vegan food and cross-contamination in the preparation of the food.^225^ The *Casamitjana Costa* case is likely to be cited persuasively with Mr Knauff’s lawyer, Wade Poziomka, quoted as saying:

[It] demonstrates to the HRTO that ethical veganism as a creed is not necessarily something novel. Ontario is not being asked to lead the way in respect of this issue—we’re simply asking the Tribunal to apply the facts of Adam’s particular case and his particular belief-system to the already-accepted creed standard in Ontario. This case shows it is already happening elsewhere.^226^

The development of the law around philosophical beliefs or creeds demonstrate that ethical veganism can be protected pursuant to anti-discrimination legislation. Indeed, Mr Knauff is hoping the ruling in his case ‘could be influential in other provinces and internationally.’^227^ The legal profession within Canada also acknowledge the development in this area with one lawyer commenting:

Times are changing. While the development of creed as a protected ground is in its infancy, we fully expect that this will become an important aspect of human rights protections in British Columbia in the near future.^228^

## Should Veganism and Vegetarianism be a Philosophical Belief?

This article has so far outlined the law relating to discrimination and protected philosophical beliefs, and the different kinds of veganism and vegetarianism, and their influencing factors. The following section analyses and discusses whether they should in fact be protected philosophical beliefs and, if so, to what extent.

The authors concur with the decision *in Casamitjana Costa* to the extent that ethical veganism should be protected as a philosophical belief. Further, the authors argue that ethical vegetarianism should also be considered a philosophical belief. What becomes more difficult is where we draw the line with what kinds of vegans and vegetarians benefit from the above law. Schwartz argues that this is dependent on how the ethical vegan/vegetarian lives their life, and how they present their arguments.^229^ Though the practice of veganism may be similar between the different groups outlined above, it is the underlying belief of the practice which is philosophical, or religious as some argue, in nature.^230^ This is a difficulty Schwartz outlines, for example, what position does a court take for a health vegan turned ethical vegan? We know from the studies discussed above that some of those who began as a vegan other than ethical, have since done research and moved over to the ethical branch. Would this restrict their claim of it being a protected philosophical belief, or even religion? The authors argue not, as the reason for starting the practice is irrelevant to the beliefs held at the time of the discrimination, and the seriousness with which they are held. It will be based on the individual and how they practice and hold that belief, as long as they maintain that it is a philosophy, which influences how they live their life and the choices they make. It is useful at this point to make an analogy with religion. It is clear that an individual who has found, or converted to religion, whatever that religion may be, would be protected from discrimination on the grounds of that religion.

Whilst the case for ethical veganism appears to be well established, should other types of veganism warrant protection as a philosophical belief? The case of *Casamitjana Costa* highlights how the Claimant lived in a way which held the belief of ethical veganism, linked to a philosophical belief that we should not harm animals, closely linked to Ahimsa philosophy. He refused to consume any food which he believed had harmed animals, but beyond this, he would not live with companion animals, and would avoid travelling on public transport which could potentially harm insects or birds. It is clear that his ethical veganism was a belief which was found to have cogency, seriousness, cohesion and importance. The authors argue that this should be the same principles are applicable for environmental vegans, with support from *Grainger plc and others v Nicholson,*^231^ which held that climate change is a protected philosophical belief. Health vegans, on the other hand, practising veganism solely for intrinsic reasons and to benefit their own health, should not be protected under law.

The authors posit that health vegans would not satisfy the criteria for establishing a philosophical belief. Firstly, it is an opinion and viewpoint that a vegan diet is healthier than a non-vegan diet. By definition, if an individual is adopting a diet for health reasons, if there was evidence to suggest an alternative non-vegan diet was healthier, it is likely their opinion and viewpoint would change. At the very least, they would consider the alternative. Secondly, the authors suggest that health veganism is a lifestyle choice that cannot be described as relating to a weighty and substantial aspect of human life and behaviour. Finally, health veganism is entirely intrinsic and therefore, in the authors’ view, lacks the necessary level of cogency, seriousness, cohesion and importance. If health veganism were considered a philosophical belief, then the belief in attending the gym might also be considered a philosophical belief. For the same reasons, the authors suggest that health veganism does not have a similar status or cogency to a religious belief.

The issue with *Casamitjana Costa* being the first case to state ethical veganism as a protected philosophical belief, is just how extremely the Claimant follows the practices and the extents he goes to, to ensure that he is not participating in the harm of animals. For future cases, it is not sure how strict a vegan has to be, or how much impact they let it have on, or influence in, their life. In *Conisbee*, for example, though the Claimant held a belief that it was wrong to kill and eat other animals, it was not found strong enough to be held as a philosophical belief, but rather an opinion or a view point. The authors believe the Employment Tribunal adopted a simplistic definition of vegetarianism and, as discussed in this article, there are many different branches of vegans and vegetarians with different influencing factors. It has already been argued that ‘at best *Conisbee* could be described as a muddled judgment; at worst, it is seriously flawed.^232^ Many vegetarians would strongly disagree that their belief is a lifestyle choice and not a philosophical belief. Further, as highlighted above, studies have shown that a number of vegans become vegetarian first before making the transition, so to deny that they have that philosophical belief whilst going on that journey is an incorrect and narrow view. As Edge stressed, the view of ethical vegetarianism that killing and eating animals is morally wrong, is wider than the decision in *Hashman v Milton Park (Dorset) Ltd*, that animals should not be hunted for sport.^233^ There needs to be a middle ground, and ethical vegetarianism, or even environmental vegetarianism, should fall on the side of protection. We can again draw an analogy with religion and consider the comparison between devout and non-devout followers of a religion. Whilst their practice, or the manifestation of the religion, may be different, the underlying belief is the same. If we consider the application of this in *Conisbee* and *Casamitjana Costa*, whilst the manifestation of their belief was significantly different, they both believed it was morally wrong to kill animals.

The authors suggest that it is misconceived to determine whether an individual has a philosophical belief based upon the label of being a vegan or a vegetarian. Whilst many vegans are likely to satisfy the criteria to qualify as a philosophical belief, some vegans may not. Similarly, some vegetarians may satisfy the criteria whilst others will not. In essence, the court or tribunal need to consider the underlying rationale as to why an individual practices veganism or vegetarianism to determine whether it is a philosophical belief, not merely looking at the manifestation of their veganism or vegetarianism. The classifications and subsets of veganism and vegetarianism may assist in this determination but ultimately, each belief is personal.

Whilst we can criticise the approach adopted by the tribunal, *Casamitjana Costa* demonstrates that ethical veganism has been judicially recognised as a philosophical belief, building upon the jurisprudence of the EHCR. *Casamitjana Costa* provides persuasive authority that ethical veganism should be a protected belief not only in other cases within England and Wales, but also in other jurisdictions. There is a distinct challenge in veganism and vegetarianism being recognised as a religion, as illustrated in the United States. However, where non-secular beliefs are protected independently of religious beliefs, there is a stronger argument for inclusion. The wording of legislation protecting non-secular beliefs may take different forms; ‘thought and conscience’; ‘philosophical beliefs’; or ‘creed’. After all, the language used is synonymous.

However, the mere recognition of ethical veganism as a protected belief does not necessarily mean significant changes in the protection afforded. The extent of protection varies across jurisdictions in the areas subject to protection on the grounds of religion and belief, as well as the types of discrimination afforded protection. The authors have identified the broad range of discrimination experienced by vegans and vegetarians, often as a consequence of the manifestation of their beliefs. There will therefore be a continuing question as to how far it is reasonable to protect or accommodate those beliefs.

## Conclusion

What has become clear throughout this article is that there is no clear definition of a vegan or a vegetarian, and what influences the choice to abstain from meat, and possibly other animal derived products, is not necessarily based on one factor. There are potentially multiple reasons as to why an individual chooses to practice veganism or vegetarianism, which can change and evolve over time. What this does mean, however, is that it can impact on whether it is perceived to be a protected philosophical belief, worthy of protection of the law.

In *Casamitjana Costa* it was clear that the Claimant has a deeply rooted philosophical belief in his veganism, to the extent that it impacted on almost every aspect of his life. What is concerning, though, is how this extremity will affect future claims and the extent a claim will have to go to, in order to justify their veganism or vegetarianism as a philosophical belief. This is exactly the issues faced in the previous case of *Conisbee*, where his vegetarianism was deemed to be a lifestyle choice, rather than appreciating that it also ‘expresses the conviction that to be fully human is to have reverence for all life, especially sentient life. This includes a rejection of violence.’^234^

Whilst the judgment in *Casamitjana Costa* is a First Instance decision, and therefore not binding, it is important. It has sent a message that the philosophical belief in ethical veganism, which has been justified through research and academic writings, is recognised, and can be protected from discrimination. The comparison to religion is not one the authors think is a substantial argument, and it is important to recognise and protect beliefs which are secular, and more aligned to philosophical beliefs or creeds. Rather than try to fit veganism and vegetarianism into the narrow definition of religion, or even argue for the establishment of a Church of Animal Liberation,^235^ those who hold this belief can still be adequately protected and realised by law.

Veganism and vegetarianism are genuine philosophical beliefs which, to the individuals who believe and practice it, is important and impacts greatly on their everyday life. This belief is strong, but not only for ethical vegans, it is also present in ethical and environmental vegans and vegetarians. The criteria and practices for establishing this belief should not be held to the high standard provided in *Casamitjana Costa,* and extended to others who hold the belief, though they may not practice it as extremely as what has been established in law. The belief that is held philosophically needs to be established in law, so that those who practice it can be protected from discrimination in law.

## Footnotes

1 Mintel Press Office, *#Veganuary: UK Overtakes Germany as World’s Leader for Vegan Food Launches*, (January 10th 2019) https://www.mintel.com/press-centre/food-and-drink/veganuary-uk-overtakes-germany-as-worlds-leader-for-vegan-food-launches.

2 Vegan Trade Journal, *Almost Half of UK Vegans Made the Change in the Last Year, According to New Data* (19th November 2018) https://www.vegantradejournal.com/almost-half-of-uk-vegans-made-the-change-in-the-last-year-according-to-new-data/.

3 Finder, *UK Diet Trends 2020* (27 April 2020) https://www.finder.com/uk/uk-diet-trends.

4 Agricultural and Horticulture Development Board, *Consumer Insights*, (July 2018) https://media.ahdb.org.uk/media/Default/Consumer%20and%20Retail%20Insight%20Images/PDF%20articles/ConsumerInsights%20WEB_1653_180725.pdf.

5 Matthew Calarco, Being toward meat: anthropocentrism, indistinction, and veganism, 38 Dialect Anthropol 415, 426 (2014).

6 Cara C. MacInnis and Gordon Hodson, It ain’t easy eating greens: Evidence of bias toward vegetarians and vegans from both source and target, 20(6) Group Processes and Intergroup Relations 721 (2017).

7 Kelsey Steele, The Vegan Journey: An Exploration of Vegan Experiences with Vegan from Burlington, Vermont, Environmental Studies Electronic Thesis Collection 23, University of Vermont (2013).

8 Kelsey Steele, The Vegan Journey: An Exploration of Vegan Experiences with Vegan from Burlington, Vermont, Environmental Studies Electronic Thesis Collection 23, University of Vermont (2013); Anna Lindquist, Beyond Hippies and Rabbit Food: The Social Effects of Vegetarianism and Veganism Honors Program Theses, University of Puget Sound (2013).

9 Anna Lindquist, Beyond Hippies and Rabbit Food: The Social Effects of Vegetarianism and Veganism Honors Program Theses, University of Puget Sound (2013); Lisa Johnson, The Religion of Ethical Veganism, 5(1) Journal of Animal Ethics 31 (2015); Swinder Janda and Phillip J. Trocchia, Vegetarianism: Toward a Greater Understanding, 18(12) Psychology and Marketing 1205 (2001).

10 Carol J. Adams, The Sexual Politics of Meat: A Feminist-Vegetarian Critical Theory, xxxvii (Bloomsbury Academic 2015).

11 Id. 142.

12 Cara C. MacInnis and Gordon Hodson, ‘It ain’t easy eating greens: Evidence of bias toward vegetarians and vegans from both source and target’ 20(6) Group Processes and Intergroup Relations 721, 726 (2017).

13 Id. 726–727.

14 Donna D. Page, Veganism and Sincerely Held Religious Beliefs in the Workplace: No Protection without Definition, 7 U. Pa. J. Lab. & Emp. L. 363 (2005).

15 Oscar Horta, ‘Discrimination Against Vegans’ 24 Re Publica 359, 370 (2018).

16 *Casamitjana Costa v The League Against Cruel Sports* [2020] ET 3331129/2018 (England and Wales).

17 *Conisbee v Crossley Farms Limited and others* [2019] ET 3335357/2018 (England and Wales).

18 United Nations Universal Declaration of Human Rights art.18.

19 The Treaty on European Union art. 6 (as amended by Treaty of Lisbon).

20 European Court of Human Rights, Guidance on Article 9 of the European Convention on Human Rights: Freedom of thought, conscience and religion para. 14 (30 April 2020) https://www.echr.coe.int/Documents/Guide_Art_9_ENG.pdf.

21 Id.

22 Id. para 17.

23 Id. paras 21–22.

24 The Equality Act 2010 consolidated discrimination and equality legislation that had developed over a period of more than 40 years. Prior to the Equality Act 2010, religion and belief was protected under The Employment Equality (Religion and Belief) Regulations 2003. The Employment Equality (Religion and Belief) Regulations 2003 were introduced pursuant to the United Kingdom’s obligations under Council Directive (EC) 2000/78.

25 Human Rights Act 1998 section 7.

26 Id, section 3.

27 Equality Act 2010, Explanatory Notes, para.51; see also Harron v Chief Constable of Dorset Police [2016] IRLR 481 (England and Wales).

28 Equality Act 2010, section 10(1).

29 Equality and Human Rights Commission, Religion or belief discrimination: Advice and Guidance https://www.equalityhumanrights.com/en/advice-and-guidance/religion-or-belief-discrimination; see also Equality Act 2010, Explanatory Notes, para.51.

30 Equality Act 2010, Explanatory Notes, para.52.

31 Id.

32 Grainger plc and others v Nicholson [2010] I.C.R. 360, 370 (England and Wales); Regulation 2 of The Employment Equality (Religion or Belief) Regulations 2003 defined ‘religion or belief’ as ‘any religion, religious belief, or similar philosophical belief.’ {emphasis added} As such, under the Regulations, a ‘philosophical belief’ would need to have some similarity to the definition of religion or religious belief to gain protection under the Regulations. The Regulations were amended by section 77(1) of the Equality Act 2006 to remove the word ‘similar’. As explained by Baroness Scotland, it is not clear whether the word ‘similar’ adds to the definition describing it as ‘redundant’ as ‘any philosophical belief must attain a certain level of cogency, seriousness, cohesion and importance, must be worthy of respect in a democratic society and must not be incompatible with human dignity.’ (HL Deb 13 July 2005, vol 673, col 1109–1110).

33 See Grainger plc and others v Nicholson [2010] I.C.R. 360, 369–370 (England and Wales).

34 U.S. Const. amend 1. art 1.

35 42. U.S.C. §2000e-2.

36 42. U.S.C. §2000e-2(j).

37 EEOC, Compliance Manual Section 12: Religious Discrimination (22 July 2008) https://www.eeoc.gov/laws/guidance/section-12-religious-discrimination.

38 Id.

39 Id.

40 906 F. Supp. 1494 (D. Wyo. 1995).

41 Id. 1502.

42 Id. 1502–1503.

43 Id. 1503.

44 Id. 1504.

45 Id.

46 Bruce Friedrich, The Church of Animal Liberation: Animal Rights as Religion under the Free Exercise Clause, 21 Animal L. 65 (2014).

47 Constitution Act, 1982, Canadian Charter of Rights and Freedoms, section 2.

48 Richard Moon, Religious Commitment and Identity: Syndicat Northcrest v. Amselem, 29(1) The Supreme Court Law Review: Osgoode’s Annual Constitutional Cases Conference 201, 214 (2005).

49 R.S.C., 1985, c. H-6, section 3(1).

50 A useful summary is provided in Ontario Human Rights Commission, Human rights and creed research and consultation report, 48 (2013) http://a.ohrc.on.ca/sites/default/files/consultation%20report_creed%20human%20rights%20research%20and%20consultation%20report.pdf.

51 Syndicat Northcrest v. Amselem [2004] 2 SCR 551 para.39 (Canada).

52 Id.

53 Id.

54 Ontario Human Rights Commission, Human rights and creed research and consultation report, 46 (2013) http://a.ohrc.on.ca/sites/default/files/consultation%20report_creed%20human%20rights%20research%20and%20consultation%20report.pdf.

55 Ontario Human Rights Commission, Policy on preventing discrimination based on creed, (17 September 2015) http://www.ohrc.on.ca/en/policy-preventing-discrimination-based-creed.

56 Id.

57 The Ontario Human Rights Commission did not incorporate a factor similar to being ‘worthy of respect in a democratic society, compatible with human dignity and not conflict with the fundamental rights of others’, whilst it is not necessary for a philosophical belief to have ‘some “nexus” or connection to an organization or community that professes a shared system of belief’.

58 Equality Act 2010 Part 3.

59 Id. Part 4.

60 Id. Part 5.

61 Id. Part 6.

62 Id. Part 7.

63 42. U.S.C. §2000e(b).

64 Section 5.

65 Section 6.

66 Section 7.

67 Section 13(1) of the Equality Act 2010 defines direct discrimination as ‘A person (A) discriminates against another (B) if, because of a protected characteristic, A treats B less favourably than A treats or would treat others.’

68 European Court of Human Rights, Guidance on Article 9 of the European Convention on Human Rights: Freedom of thought, conscience and religion para. 25 (30 April 2020) https://www.echr.coe.int/Documents/Guide_Art_9_ENG.pdf.

69 ECHR art. 9(2).

70 Equality Act 2010 section 19(1) and (2)(a)–(c).

71 Id. section 19(2)(d).

72 *Wasteney v East London NHS Foundation Trust* UKEAT/0157/15/LA (England and Wales).

73 Equality Act 2010 section 26(1).

74 Similar to direct discrimination.

75 Similar to indirect discrimination.

76 42. U.S.C. §2000e-2(j).

77 Included within the definition of disparate treatment.

78 Sections 5–9.

79 Section 14.

80 Section 14.1.

81 Section 10.

82 Peter Singer, Animal Liberation (Random House 1975).

83 Tom Regan, The Case for Animal Rights (University of California Press 1983).

84 Gary L. Francione, *Some Thoughts on the Meaning of Vegan,* Abolitionist Approach (18 October 2009) https://www.abolitionistapproach.com/some-thoughts-on-the-meaning-of-vegan/.

85 Id.

86 Gary L. Francione, Reflections on Animals, Property, and the Law and Rain without Thunder, 70 Law & Contemp. Probs. 9 (2007).

87 Gary L. Francione, Animal Rights: The Abolitionist Approach, 224–28 (Exempla Press 2015).

88 Kelsey Steele, ‘The Vegan Journey: An Exploration of Vegan Experiences with Vegan from Burlington, Vermont’ Vermont, Environmental Studies Electronic Thesis Collection 23, University of Vermont, 15 (2013).

89 Christopher Key Chapple, Nonviolence to Animals, Earth and Self in Asian Traditions 10 (State University of New York Press, 1993).

90 Kavita Bhatt, ‘Ahimsa: The Jain’s Strategy for the Conservation of Biodiversity’, as found in Indoo Pandey Khanduri (Ed) Human Freedom and Environment: Contemporary Paradigms and Moral Strategies 302 (Kalpaz Publications, 2010).

91 Steven L. Davis, The Least Harm Principle May Require that Humans Consume a Diet Containing Large Herbivores, Not a Vegan Diet, as found in S. Armstrong and R. Botzler (Eds), The Animal Ethics Reader (3rd edn, Routledge 2016).

92 Id. 267.

93 Gary L. Francione, Animal Rights: The Abolitionist Approach (Exempla Press 2015).

94 *Casamitjana Costa v The League Against Cruel Sports* [2020] UK ET 3331129/2018, para 17 (England and Wales).

95 The Vegan Society, *Definition of Veganism*, https://www.vegansociety.com/go-vegan/definition-veganism.

96 Leah Leneman, No Animal Food: The Road to Veganism in Britain, Britain 7(3) Society and Animals 219, 221 (1999).

97 Id. 222.

98 Id.

99 David DeGrazia, Meat Eating, as found in S. Armstrong and R. Botzler (Eds), The Animal Ethics Reader (3rd edn, Routledge 2016).

100 Marian Stamp Dawkins, Why We Still Need to Read Animal Machines, as found in Ruth Harrison, Animal Machines, 2 (First published 1964, 2013 CABI).

101 Ruth Harrison, Animal Machines (First published 1964, 2013 CABI).

102 The current legislation is the Welfare of Farmed Animals (England) Regulations 2000.

103 Council Directive 2008/119/EEC.

104 Henning Steinfeld et al., Livestock’s Long Shadow: Environmental Issues and Options, 7–11 (Food & Agriculture Org 2006).

105 Id. 11–20.

106 Compassion in World Farming, Strategic Plan 2018–2022: Working Together to End Factory Farming Worldwide https://assets.ciwf.org/media/7432824/ciwf_strategic-plan-revise18-lr2.pdf.

107 Ruth Harrison, Animal Machines (First published 1964, 2013 CABI).

108 Peter Singer, Animal Liberation (Random House 1975).

109 Compassion in World Farming, *Farm Animals: Chickens*, < https://www.ciwf.org.uk/farm-animals/chickens/> accessed 5.06.20.

110 It should be noted that these statistics were recorded during the Covid-19 pandemic and may not be as representative of how many animals are slaughtered due to the issues faced by farmers.

111 Department for Environment, *Food and Rural Affairs, United Kingdom Slaughter Statistics—April 2020* (14th May 2020) https://assets.publishing.service.gov.uk/government/uploads/system/uploads/attachment_data/file/884944/slaughter-statsnotice-14may20.pdf.

112 Department for Environment, Food and Rural Affairs, *United Kingdom Poultry and Poultry Meat Statistics—April 2020 (*21st May 2020) https://assets.publishing.service.gov.uk/government/uploads/system/uploads/attachment_data/file/886407/poultry-statsnotice-21may20.pdf.

113 It must be noted that battery cages are now banned in some parts of the world. For example, the EU effectively banned battery cages in 2012 through Directive 1999/74/EC. They are still used in the USA, however. For more information, please see: Jessica Braunschweig-Norris, The U.S. Egg Industry - Not All It's Cracked up to Be for the Welfare of the Laying Hen: A Comparative Look at United States and European Union Welfare Laws, 10 Drake J. Agric. L. 511 (2005).

114 Whitney R. Morgan, Proposition Animal Welfare: Enabling an Irrational Public or Empowering Consumers to Align Advertising Depictions with Reality, 26 U. Fla. J.L. & Pub. Pol'y 297 (2015).

115 Id.

116 Compassion in World Farming, *Farm Animals: Chickens Farmed for Meat* https://www.ciwf.org.uk/farm-animals/chickens/meat-chickens/.

117 The Vegan Society, *The Honey Industry* https://www.vegansociety.com/go-vegan/why-go-vegan/honey-industry.

118 For example, please see, Rachel Dunn, For Fur’s Sake: Can the UK Can Imports of Fur from Other Countries?, 3(1) The UK Journal of Animal Law 42 (2019).

119 For example, please see, Peter Singer, Animal Liberation (Random House 1975).

120 For example, please see, Trevor J. Smith, Bullhooks and the Law: Is Pain and Suffering the Elephant in the Room? 19(2) Animal Law Review 423 (2013).

121 David DeGrazia, Meat Eating, as found in S. Armstrong and R. Botzler (Eds), The Animal Ethics Reader 428 (3rdedn, Routledge 201).

122 Pew Commission, *Environmental Impact of Industrial Farm Animal Production: A Report of the Pew Commission on Industrial Farm Animal Production* https://www.lclark.edu/live/files/6699-environmental-impact-of-industrial-farm-animal.

123 Liam H. Michener, Meating America's Demand: An Analysis of the Hidden Costs of Factory Farming and Alternate Methods of Food Production, 7 J. Animal & Envtl. L. 145 (2015).

124 P. Gerber, C. Opio and H. Steinfeld, Poultry production and the environment*—*a review, Animal Production and Health Division, Food and Agriculture Organization of the United Nations, 3 http://www.fao.org/ag/againfo/home/events/bangkok2007/docs/part2/2_2.pdf.

125 Sara J. Scherr and Satya Yadav, Land Degradation in the Developing World: Implications for Food, Agriculture, and the Environment to 2020, Food, Agriculture, and the Environment Discussion Paper 14, 3 (1996).

126 Id. 7.

127 Henning Steinfeld *et al*, Livestock’s Long Shadow: Environmental Issues and Options 29 (Food & Agriculture Org 2006).

128 Leo Horrigan, Robert S. Lawrence and Polly Walker, How Sustainable Agriculture Can Address the Environmental and Human Harms of Industrial Agriculture, 110(5) Environmental Health Perspectives 445, 448 (2002).

129 Compassion in World Farming, *Beyond Factory Farming: Sustainable Solutions for Animals, People and the Planet* 39 (2009) https://www.compassioninfoodbusiness.com/media/3817096/beyond-factory-farming-report.pdf.

130 Bingli Clark Chai *et al*, Which Diet has the Least Environmental Impact on our planet? A Systematic Review or Vegan, Vegetarian and Omnivorous Diets, 11(15) Sustainability 4410 (2019).

131 David DeGrazia, Meat Eating, as found in S. Armstrong and R. Botzler (Eds), The Animal Ethics Reader 428 (3rdedn, Routledge 2016).

132 Compassion in World Farming, *Beyond Factory Farming: Sustainable Solutions for Animals, People and the Planet* 36 (2009) https://www.compassioninfoodbusiness.com/media/3817096/beyond-factory-farming-report.pdf.

133 Christian Nellemann *et al*, The Environmental Food Crisis: The Environment’s Role in Averting Future Food Crises, 27 (2009) https://www.gwp.org/globalassets/global/toolbox/references/the-environmental-crisis.-the-environments-role-in-averting-future-food-crises-unep-2009.pdf.

134 Leo Horrigan, Robert S. Lawrence and Polly Walker, How Sustainable Agriculture Can Address the Environmental and Human Harms of Industrial Agriculture, 110(5) Environmental Health Perspectives 445, 451 (2002).

135 Ian Phillips *et al*, Does the use of antibiotics in food animals pose a risk to human health? A critical review of published data, 53(1) Journal of Antimicrobial Chemotherapy 28 (2004).

136 World Health Organisation, *Stop using antibiotics in healthy animals to prevent the spread of antibiotic resistance* (7 November 2017) https://www.who.int/news-room/detail/07-11-2017-stop-using-antibiotics-in-healthy-animals-to-prevent-the-spread-of-antibiotic-resistance.

137 Regulation 1831/2003/EC.

138 Brigit Lassok and Bernd-Alois Tenhagen, From Pig to Pork: Methicillin-Resistant *Staphylococcus aureus* in the Pork Production Chain, 76(6) Journal of Food Production 1095 (2013).

139 Stephen S Morse *et al*, Prediction and prevention of the next pandemic zoonosis, 380(9857) Lancet 1956 (2012).

140 L.F. Wang and G. Crameri, Emerging zoonotic viral diseases, 33(2) Rev SciTech Off Int Epiz 569 (2014).

141 For example, please see Jimmy Whitworth, COVIDCOVID-19: A fast evolving pandemic, 114(4) Transactions of the Royal Society of Tropical Medicine and Hygiene 241(2020).

142 Stephen S Morse *et al*, Prediction and prevention of the next pandemic zoonosis, 380(9857) Lancet 1956 (2012).

143 FOA Animal Production and Health Paper, *Improved animal health for poverty reduction and sustainable livelihoods* 19 (2002) http://www.fao.org/3/a-y3542e.pdf.

144 Michael Greger, The Human/Animal Interface: Emergence and Resurgence of Zoonotic Infectious Diseases, 33(4) Critical Reviews in Microbiology 243 (2007).

145 Alessandra Petti *et al*, Vegetarianism and veganism: not only benefits but also gaps. A Review, 19(3) Progress in Nutrition 229, 231 (2017).

146 F Phillips, Vegetarian nutrition, British Nutrition Foundation: Briefing Paper (2005).

147 Elizabeth Anne Jessie Cook *et al*, Working conditions and public health risks in slaughterhouses in western Kenya, 17 BMC Public Health (2017).

148 Jennifer Dillard, A Slaughterhouse Nightmare: Psychological Harm Suffered by Slaughterhouse Employees and the Possibility of Redress through Legal Reform, 15 Geo. J. on Poverty L. & Pol'y 391 (2008).

149 Gail A. Eisnitz, Slaughterhouse: The Shocking Story of Greed, Neglect, and Inhumane Treatment Inside the US Meat Industry (Prometheus Books 2006).

150 Stephanie Marek Muller, Zombification, Social Death, and the Slaughterhouse: U.S. Industrial Practices of Livestock Slaughter, 53(3) American Studies 81 (2018).

151 BBC News: Stories, *Confessions of a slaughterhouse worker* (6th January 2020) https://www.bbc.co.uk/news/stories-50986683.

152 Karen Victor and Antoni Barnard, Slaughtering for a living: A Hermeneutic phenomenological perspective on the well-being of slaughterhouse employees, 11 International Journal of Qualitative Studies in Health and Well-being (2016).

153 Jennifer Dillard, A Slaughterhouse Nightmare: Psychological Harm Suffered by Slaughterhouse Employees and the Possibility of Redress through Legal Reform, 15 Geo. J. on Poverty L. & Pol'y 391 (2008).

154 Oxfam Research Report, *Lives on the Line: The Human Cost of Cheap Chicken* (2015) https://s3.amazonaws.com/oxfam-us/www/static/media/files/Lives_on_the_Line_Full_Report_Final.pdf.

155 Stephanie Marek Muller, Zombification, Social Death, and the Slaughterhouse: U.S. Industrial Practices of Livestock Slaughter, 53(3) American Studies 81, 82 (2018).

156 Elizabeth Cherry, Veganism as a Cultural Movement: A Relational Approach, 5(2) Social Movement Studies 155, 156 (2006).

157 Jessica Greenebaum, Veganism, Identity and the Quest for Authenticity, 15(1) Food, Culture and Society 129 (2012).

158 For example, Lisa Johnson, The Religion of Ethical Veganism, 5(1) Journal of Animal Ethics 31 (2015).

159 Rebecca Schwartz, Employers, Got Vegan: How Ethical Veganism Qualifies for Religious Protection under Title VII, 24 Animal L. 221, 224 (2018).

160 Please see David Newton, Vegetarianism and Veganism: A Reference Handbook (Contemporary World Issues) (ABC-CLIO 2019) 38–41; Leah Leneman, No Animal Food: The Road to Veganism in Britain, 7(3) Society and Animals 219 (1999).

161 Id. 40.

162 Swinder Janda and Phillip J. Trocchia, Vegetarianism: Toward a Greater Understanding, 18(12) Psychology and Marketing 1205 (2001).

163 Malcolm Hamilton, Eating Ethically: ‘Spiritual’ and’Quasi-Religious’ Aspects of Vegetarianism, 15(1) Journal of Contemporary Religion 65, 69 (2000).

164 Lyle Munro, Strategies, Action Repertoires and DIT Activism in the Animal Rights Movement, 4(1) Social Movement Studies 75, 76 (2005).

165 Jessica Greenebaum, Veganism, Identity and the Quest for Authenticity, 15(1) Food, Culture and Society 129, 130 (2012).

166 Id. 134.

167 Jain Maxim, Vegan Values, Religious Rights: A Cultural Critique of Entrenched Ethics, Conference paper Lewis & Clark Animal Law Conference, 5 (October 2010).

168 Sarah E. Mann, More Than Just A Diet: An Inquiry Into Veganism, Paper 156 Anthropology Senior Theses, 59 (2014).

169 Id. 60.

170 Id. 62.

171 Id. 70.

172 Laura Jennings *et al*, Exploring Perceptions of Veganism, (2019) https://arxiv.org/pdf/1907.12567.pdf.

173 *W v the United Kingdom* (App No 18187/91) Commission Decision 10 February 1993; see also European Court of Human Rights ‘Guide on Article 9 of the European Convention on Human Rights: Freedom of thought, conscience and religion’ (30 April 2020), para.17(c); This case concerned a vegan prisoner who objected to working in a print shop because his beliefs prevented him from working with products tested on animals (i.e. the dyes). It should be noted that the United Kingdom Government did not contest whether veganism was protected under Article 9 ECHR. The case was determined on the basis that the applicant had not exhausted his domestic remedies.

174 ‘Vegetarianism’ has been cited as an ‘uncontroversial’ example of a belief that would fall within the scope of Article 9 See *Regina v Secretary of State for Education and Employment & Others (Respondents) ex parte Williamson (Appellant) & Others* [2005] UK HL 15, para.55 (England and Wales).

175 *Hashman v Milton Park (Dorset) Limited t/a Orchard Park* [2011] ET 3105555/2009 (England and Wales); the case concerned the definition of ‘philosophical belief’ within the context of the Employment Equality (Religion or Belief) Regulations 2003.

176 Id. para. 55; the case particularly related to the Claimant’s belief in anti-hunt activism.

177 House of Lords Debate, UK, 23 March 2010, vol 718, col 853.

178 Equality and Human Rights Commission: Draft Code of Practice Equality Act as stated Practical Law Employment: Religion or Belief Discrimination; See also House of Lords Debate, UK, 23 March 2010, vol 718, col 852–853.

179 Practical Law Employment: Religion or Belief Discrimination.

180 *Conisbee v Crossley Farms Limited and others* [2019] ET 3335357/2018 (England and Wales).

181 The Claimant alleged that a colleague had called him ‘gay’ because he was a vegetarian (para.56) whilst he was also shouted at in front of customers - *Conisbee v Crossley Farms Limited and others* [2019] ET 3335357/2018 (England and Wales) para.7.

182 *Conisbee v Crossley Farms Limited and others* [2019] ET, para.39.

183 Id. para.38.

184 Id. para.42.

185 Id. para.39.

186 Id. para.40.

187 Id. para.41.

188 Id. para.43.

189 *Casamitjana Costa v The League Against Cruel Sports* [2020] ET 3331129/2018 (England and Wales).

190 Jordi Casamitjana, *Help an Ethical Vegan who was dismissed by an Animal Welfare charity*, www.crowdjustice.com/case/help-a-discriminated-ethical-vegan/.

191 Id.

192 The authors have no knowledge of an appeal being lodged in the case of *Conisbee* whilst the parties settled in *Casamitjana Costa* (Casamitjana Costa v The League Against Cruel Sports [2020] UK ET 3331129/2018 Consent Judgment (England and Wales)).

193 *Casamitjana Costa v The League Against Cruel Sports* [2020] ET 3331129/2018 (England and Wales) para.20.

194 Id.

195 Incidentally, both *Conisbee* and *Casamitjana Costa* were heard before the same Employment Judge.

196 *Conisbee v Crossley Farms Limited and others* [2019] ET 3335357/2018 (England and Wales) para.39.

197 *Casamitjana Costa v The League Against Cruel Sports* [2020] ET 3331129/2018 (England and Wales) para.34.

198 *McClintock v Department of Constitutional Affairs* UKEAT/0223/07/CEA (England and Wales).

199 Id. para.45.

200 *Conisbee v Crossley Farms Limited and others* [2019] ET 3335357/2018 (England and Wales) para.16b.

201 *Hashman v Milton Park (Dorset) Limited t/a Orchard Park* [2011] ET 3105555/2009 (England and Wales).

202 Id. para.40.

203 *Grainger plc and others v Nicholson* [2010] I.C.R. 360, 371 (England and Wales).

204 Id.

205 *Casamitjana Costa v The League Against Cruel Sports* [2020] ET 3331129/2018 (England and Wales) para.35.

206 *Conisbee v Crossley Farms Limited and others* [2019] ET 3335357/2018 (England and Wales) para.41.

207 Id.

208 Id.

209 Id.

210 *Casamitjana Costa v The League Against Cruel Sports* [2020] ET 3331129/2018 (England and Wales) para.36.

211 *Conisbee*, *v Crossley Farms Limited and others* [2019] ET 3335357/2018 (England and Wales) para.43.

212 *Casamitjana Costa v The League Against Cruel Sports* [2020] ET 3331129/2018 (England and Wales) para.11.

213 Rebecca Schwartz, Employers, Got Vegan: How Ethical Veganism Qualifies for Religious Protection under Title VII, 24 Animal L. 221, 230 (2018).

214 No. B150017. Second Dist., Div. Five. Sept. 13, 2002.

215 *McDavid v County of Sacramento* ED Cal., June 27, 2006.

216 *Anderson v Orange County Transit Authority*, Charge No. 345960598, EEOC Determination Letter.

217 *Chenzira v Cincinnati Childrens' Hospital*, No. 1:2011cv00917 - Document 18 (S.D. Ohio 2012).

218 2012 HRTO 994 (Ontario, Canada).

219 Id. para.2.

220 Id.

221 Labchuck, C., Protecting secular beliefs: Should creed provisions protect ethical vegans from discrimination?, Paper presented at the Ontario Human Rights Commission/York University Legal Workshop on Human rights, creed and freedom of religion (2012, March 29–30) Osgoode Hall, York University. Retrieved from https://docs.google.com/file/d/0BwFvhg37TTCjMk84Q0l4QTdic3M/preview; Sztybel, D., Giving credence to philosophical creeds: The cases of Buddhism and veganism. Paper presented at the Ontario Human Rights Commission/York University Legal Workshop on Human rights, creed and freedom of religion (2012, March 29–30) Osgoode Hall, York University Retrieved from https://docs.google.com/file/d/0BwFvhg37TTCjS1Awa1JSNkJZNWM/preview?pli=1 as cited in Ontario Human Rights Commission, Human Rights and Creed: Research and consultation report, 49 (2013).

222 Id.

223 Id.

224 Ontario Human Rights Commission, Policy on preventing discrimination based on creed, (17 September 2015) http://www.ohrc.on.ca/en/policy-preventing-discrimination-based-creed.

225 Veromi Arsiradam, *A Case for Recognizing Ethical Veganism as Creed,* Obiter-Dicta (10 March 2020) https://obiter-dicta.ca/2020/03/10/a-case-for-recognizing-ethical-veganism-as-creed/.

226 Id.

227 Lily Puckett, ‘*Vegan firefighter complains to Human Rights Tribunal because he wasn’t offered enough* food’*food,* Independent (22 May 2019) https://www.independent.co.uk/news/world/americas/vegan-firefighter-sue-food-ontario-human-rights-a8926246.html.

228 Richard Johnson, ‘*Creed: Protecting Veganism as a Human* Right’ *Right* (11 March 2020) https://kentemploymentlaw.com/2020/creed-protecting-veganism-as-a-human-right/.

229 Rebecca Schwartz, Employers, Got Vegan: How Ethical Veganism Qualifies for Religious Protection under Title VII, 24 Animal L. 221 (2018).

230 Lisa Johnson, The Religion of Ethical Veganism, 5(1) Journal of Animal Ethics 31 (2015).

231 *Grainger plc and others v Nicholson* [2010] I.C.R. 360, 371 (England and Wales).

232 Frank Cranmer, A Critique of the Decision in *Conisbee* that Vegetarianism in not ‘A Belief’, 22(1) Ecclesiastical Law Journal 36, 48 (2020).

233 Peter Edge, *Vegetarianism as a protected characteristic: Another view on Conisbee, Law and Religion UK* (23 September 2019) https://www.lawandreligionuk.com/2019/09/23/vegetarianism-as-a-protected-characteristic-another-view-on-conisbee/.

234 Malcolm Hamilton, Eating Ethically: ‘Spiritual’ and’Quasi-Religious’ Aspects of Vegetarianism’ (2000), 15(1) Journal of Contemporary Religion 65, 70 (2000).

235 Bruce Friedrich, The Church of Animal Liberation: Animal Rights as Religion under the Free Exercise Clause, 21 Animal L. 65 (2014) .

